# Genetics of human malignant peripheral nerve sheath tumors

**DOI:** 10.1093/noajnl/vdz049

**Published:** 2019-11-28

**Authors:** Alexander Pemov, Hua Li, William Presley, Margaret R Wallace, David T Miller

**Affiliations:** 1 Clinical Genetics Branch, Division of Cancer Epidemiology and Genetics, National Cancer Institute, National Institutes of Health, Rockville, Maryland; 2 Department of Molecular Genetics and Microbiology, University of Florida College of Medicine, Gainesville, Florida; 3 University of Florida Health Cancer Center, University of Florida, Gainesville, Florida; 4 Division of Genetics and Genomics, Boston Children’s Hospital, Boston, Massachusetts

**Keywords:** Epigenomics, genomics, MPNST, NF1, transcriptome

## Abstract

Malignant peripheral nerve sheath tumors (MPNSTs) are heterogeneous, highly aggressive tumors with no widely effective treatment other than surgery. Genomic architecture of MPNST is similar to other soft tissue sarcomas, with a relatively modest burden of single nucleotide variants and an elevated frequency of copy-number alterations. Recent advances in genomic studies identified previously unrecognized critical involvement of polycomb repressor complex 2 (PRC2) core components *SUZ12* and *EED* in transition to malignancy. Notably, somatic changes in *NF1*, *CDKN2A/B*, and PRC2 are found in most MPNST regardless of their etiology (e.g. neurofibromatosis type 1-associated vs. sporadic vs. radiation-induced), indicating that similar molecular mechanisms impact pathogenesis in these neoplasms. The timing and specific order of genetic or epigenetic changes may, however, explain the typically poorer prognosis of NF1-associated MPNSTs. Studies that reveal genes and regulatory pathways uniquely altered in malignancies are essential to development of targeted tumor therapies. Characterization of MPNST molecular profiles may also contribute to tools for earlier detection, and prediction of prognosis or drug response. Here we review the genetic discoveries and their implications in understanding MPNST biology.

**Key Points**

- Somatic changes in NF1, CDKN2A/B, and PRC2 are found in most MPNST regardless of their etiology, but MPNST are genomically complex, and the order and timing of other genetic events is still poorly understood since most of the available data is based on bulk analysis of tumor specimens.
- Like most other soft-tissue sarcomas, MPNSTs carry a relatively low burden of single nucleotide variants, but consistently display a high number of structural copy number variants that are relatively unique to each tumor.
- Studies of epigenetic alterations, such as loss of H3K27 trimethylation and consequent changes in gene expression, may facilitate a better understanding of MPNST biology.
- Ongoing research to identify correlations between MPNST molecular profiles and clinical behavior may provide more reliable diagnostic and prognostic information.

Malignant peripheral nerve sheath tumors (MPNSTs) are rare, invasive soft tissue sarcomas originating from nerve sheath cells (Schwann cells), with an incidence of 1.46/100 000.^1^ MPNST has a poor prognosis, with surgical resection considered the only highly effective clinical option. The term MPNST replaces previous names including malignant schwannoma, neurofibrosarcoma, and neurogenic sarcoma. About half of MPNSTs occur in people with neurofibromatosis type 1 (NF1), an autosomal dominant tumor predisposition syndrome with an incidence of 1 in 2500–3000 births worldwide.^2^ Retrospective studies of MPNSTs in people with NF1 reported a 5-year disease-free survival rate of 34%–60%, which is somewhat worse than sporadic MPNST (in people without NF1).^3^ NF1 results from germline heterozygosity for an *NF1* gene pathogenic variant resulting in reduced activity of the gene’s encoded protein neurofibromin, a RAS GTPase tumor suppressor. About 7% of people with NF1 bear a germline microdeletion spanning the *NF1* gene and flanking loci; there is debate about whether this confers higher risk for MPNST (reviewed by Kehrer-Sawatzki et al., 2017).^4^

Tumors and other features of NF1 are clonally initiated by somatic mutation of the remaining normal gene copy in individual cells. Plexiform neurofibromas are due to such bi-allelic *NF1* mutations in the Schwann cell lineage in major nerves, but typically otherwise do not have additional somatic driver mutations.^5^ These benign tumors occur in about 50% of people with NF1, with a 10–30% lifetime chance of transformation into MPNST via accumulation of genetic changes.^6–10^ In NF1-related MPNSTs, the somatic *NF1* mutation is often a deletion spanning most or all of the *NF1* gene (may be the entire chromosome) except in the germline microdeletion cases.^11^ Plexiform neurofibromas bearing premalignant histological characteristics are termed “atypical neurofibromas,” tend to carry deletion of the *CDKN2A/B* locus, and are at higher risk for transformation to MPNST.^8,12–14^ As described in detail below, the genetic and epigenetic abnormalities in MPNSTs have thus far been identified through cytogenetics, molecular analysis of individual genes, array comparative genomic hybridization (aCGH), RNA expression arrays, and next generation sequencing of exomes. A number of studies have also utilized the relatively limited number of MPNST-derived cell lines (those most commonly reported are listed in Table 1).

**Table 1.** MPNST cell lines in multiple research publications

Name	Original reference(s)	Subject has NF1? (gender)	Tumor	Mutations: *NF1* germline based on NM_0000267.3; *TP53* specific somatic based on NM_000546.5.
ST-8814	^78,79^	Yes (male)	n.a.	*NF1* c.910 C>T exon skip
sNF96.2^#^	^26^	Yes (male)	Primary	*NF1* c.3683delC
sNF02.2^#^	^80^	Yes (male)	Metastasis	*NF1* c.4868A>T
sNF94.3^#^	^81^	Yes (female)	Metastasis	*NF1* microdeletion
88-3	^82^	Yes (female)	Primary	*NF1* c.6952T>C *TP53* c.535C>T
88-14	^82^	Yes, recurrence of NF88-3	Primary, recurrence	*NF1* c.6952T>C *TP53* c.535C>T
90-8 (NF90-8)	^83,84^	Yes (female)	n.a.	*NF1* microdeletion *TP53* c.96 + 1G>A
T265 (T265-2c)*	^85^	Yes (n.a.)	n.a.	n.a.
S462	^86^	Yes (female)	n.a.	*TP53* c.389G>C
S520	^86^	n.a.	n.a.	n.a.
STS26T	^87,88^	No (female)	Metastasis	*TP53* homozygous deletion
S805	^89^	Yes (n.a.)	n.a.	n.a.
MPNST-642	^90^	Yes (male)	Primary, recurrence	n.a.
MPNST-724	^57^	No	n.a.	*TP53* c.280delTCA
Hs-Sch-2	^91^	No (female)	Primary	*TP53* c.818G>A
FU-SFT8611	^92^	No (male)	Metastasis	n.a.
FU-SFT8710	^92^	Yes (female)	Primary	n.a.
FU-SFT9817	^92^	No (female)	Primary, recurrence	n.a.
NMS-2	^93^	Yes (male)	Primary	n.a.
NMS-2PC	^93^	Yes, metastasis of NMS-2	Metastasis	n.a.

n.a., not available.

^#^Available in ATCC repository (atcc.org).

*Recent data suggest that T265 was overgrown by ST88-14 at a very early passage, so T265 data in the literature may not be reliable (Terribas, Gel, Serra, Wallace, Ratner, Largaespada, and others, in preparation).

## Genetic MPNST Alterations Identified by Karyotype and aCGH

Prior to next-generation sequencing, genome-level studies of MPNSTs were limited to karyotypes (from short-term cell culture) and aCGH. Karyotyping, the first genetic tool employed in MPNST, has a lower limit detection of 2–3 Mb, resolution limited to chromosome band level, cannot detect isodisomy, and is reliant on tumor cell division in culture. aCGH involves competitive hybridization of tumor DNA to arrays tiled with DNA probes across the genome, to obtain somatic copy number alteration (SCNA) information about each region: neutral, gain or loss relative to diploid. aCGH has much higher resolution than karyotyping, but cannot detect copy-neutral isodisomy (unless the array also has single nucleotide polymorphism probes) or balanced rearrangements. Another limitation is that tumor abnormalities may be masked by contaminating nontumor tissue included in the DNA preparation.

An extensive, detailed review of MPNST karyotype and aCGH studies was published in 2008, providing karyotypes and SCNA summaries.^15^ Every chromosome has been found involved in numerical or structural abnormalities in MPNSTs. Although many MPNSTs show multiple different cytogenetic aberrations and aneuploidy, a few also have been reported with a normal karyotype or lack of SCNAs. The review noted that cytogenetic abnormalities from over 100 MPNSTs were heterogeneous structurally and numerically, including ploidy (from hypodiploidy to near-tetraploidy), and with different clonal elements.^15^ There were no abnormalities specific to, or highly consistent within MPNST, although those derived from people with NF1 were more likely to show copy-number variants for chromosome 17 (including the *NF1* gene locus) or have copy-neutral losses through isodisomy. Losses at 9p21, often homozygous, were among the most common SCNAs reported, consistent with loss of the *CDKN2A/B* tumor suppressor locus in MPNST progression as mentioned above. Double minute chromosomes, often consistent with oncogene amplification, were seen in about 10% of cases based on karyotypes, although there were no reports of very high-level amplifications. Only one publication mentioned microsatellite instability, finding it present at low levels in 30% of MPNSTs.^16^Table 2 summarizes aCGH results, which together validate that MPNSTs typically contain numerous SCNAs independent of their structural rearrangements, which may be correlated with clinical outcome. More recently, approaches to detect SCNAs in MPNST have utilized next-generation sequencing with substantial read depth.^17^

**Table 2.** Array comparative genomic hybridization analysis of primary MPNSTs

Reference	# MPNSTs (NF1, non-NF1)	Findings
^22^	31 (9,22)	Two NF1 high-grade tumors had no SCNAs; others: average 11 SCNAs/tumor. Gains more common than losses, and most frequent gains were 8q23-q24.1 (12 cases), 5p14 (11 cases), 6p22-pter, 7p15-p21, 7q32-q35, 8q21.1-q22, 8q24.2-qter, and 17q22-qter (10 cases each). Eight samples (two NF1) had amplifications: 8q24-ter (three cases), and two cases each at 5p14, 7p14-pter, 8q21.1-q23, and 13q32-q33. Most common losses were 14q24.3-qter (five cases), and 1p22-p36.1, 13q21-q31, and 14q21-q24.2 (four cases each). One MPNST recurrence had a novel gain of 1p21-22. Gain of 7p15-p21 or 17q22-qter correlated with poorer survival rate. Chromosome 7 over-represented among abnormalities.
^94^	19 (6,13)	One MPNST (non-NF1) had no SCNAs; others: average 13 SCNAs/tumor. Gains more common than losses. The only amplifications were in non-NF1 (sporadic) tumors, with 5p and 12q coamplified, and other amplifications unique. Gain of 7p11-p13 and 17q24-q25 in 52% of cases. Gain of 5p15 in 47% cases. About 42% of cases had gain of 8q22-q24 or 12q21-q24. One sporadic had only 17q25 gain. One sporadic only had gain of 9q34 and 14q31q32. One sporadic only had gains 4p15p16, 5p15, 9q31q33, 12q14q22, 12q24.3, 14q31q32, and 21q22. Gains of 1q31-q32, 7q11-q31, and 9q32-q33 were each in 37% of tumors.
^95^	12 (8,4)	All had at least some SCNAs. Losses included 3p21-pter (2), 9p23-pter (4), chrom.10 (4), 11q23-qter (6), chromosome 16 or16q24 (4), chromosome 17 (2), and chromosome 22 or 22q (10). Gains included chromosome 7 or 7q (3) and chromosome 8 or 8q (2). No obvious differences between NF1 and non-NF1.
^96^	8 (5,3)	All had at least some SCNAs. No clear differences between NF1 and non-NF1. Most had amplifications, and had more gains than losses. Average 17 SCNAs/tumor. No losses common to more than two tumors except 16q12-22 in three. Gains: 4q26 and 6q (7 each), 4q12-q26, 11q14, 15q21, 21q21 (6 each), and 5p14, 5q21, 11q14-q22, 12q21, 14q21 (5 each). One had loss of chromosome 22. Specific gains found at genes *EGFR*, *MSH2*, *CDK6*, *DDX15*; specific losses were at genes *CDH1*, *EGR1*, *CTSB*, *GATA3*.
^21^	7 (4,3)	One case had no SCNAs except gain of 8q. Most frequent minimal recurrent gains: 1q24.1-q24.2, 1q24.3-q25.1, 8p23.1-p12, 9q34.11-q34.13, and 17q23.2-q25.3, in 5/7 cases (those five patients had a poor outcome, but not the other two (NF1)). Gains in *<*4 cases: 2q11.2-q13, 3p26.2-p25.1, 5q34-q35.3, 7q11.23-q21.11, 9q21.32-q22.33, 12q13.3-q15, 13q22.1-q22.2, 16p13.3-p13.2,16p13.12-p13.11, and 19p13.3-p13.2. Losses: four tumors showed loss of 11p13; 11q22.3-q23.1 and 11q23.2-q23.3 were lost in three tumors. Loss of 9p22.3-p21.2 and 14q21.3-q23.3 were observed in three cases (two homozygous and one heterozygous, all with poor survival). Loss of 11q23.2-11q23.3 was seen only in the three sporadic tumors. A few high-level amplifications were seen, predominantly on 1q and 12q.
^19^	51 (16,35)	Loss of 9p21.3 (containing *CDKN2A/B* genes) was seen in 33 cases. EGFR amplification (7q11) was seen in 19 (FISH validated). At least one EGF pathway gene was altered in 84% of samples: *EGFR* amplification (19), *GRB2* amplification (16, at 17q25), *HRAS* deletion (17, at 11p15), *MAPK1* deletion (21, at 22q11), *AKT1* deletion (16, at 14q32), *JAK2* deletion (24, at 9p24). No correlations with survival.
^97^	2 (0,2)	Gain regions common to both: 4q28-qter, 8q12-q21.1. One had multiple other gains but no losses; the other also had gain of 17q22-qter and loss of 4p14-pter.
^32^	48 (28,20)	Chromosomal and array CGH were used. Four tumors had no SCNAs; the others had median of 18 SCNAs/tumor (median 18 in NF1 tumors, 12 in sporadic tumors). Most frequent gains: 8q (in 30 cases), 17q (29), and 7p (23). Most frequent losses: 9p (30), 11q (21), and 17p (19). Most tumors had similar number of gains and losses, regardless of NF1 status. NF1 tumors more likely to have gains at 6q, 7p, 17q and losses at 4q, 11q, 13q, 18p. Most frequent amplification was 17q24.2-25.3 (in 45%). Six had homozygous deletion at 9p21.3. Worse survival was associated with tumors having gain of 16p, or loss of 10q or loss of Xq.
^98^	24 (24,0)	Gains more common than losses. Average of 12.8 SCNAs/sample. Gains seen in 15 or more tumors: 7p14, 5q14.1, 7q36.1. Most frequent loss: 9p21.3 in 8 cases. Gains in *>*9 tumors: 7p14.1-p13, 5q14.1, 7q36.1, 5q32.2, 6p22.3, 1q25.3, 3q13.11, 5q21.3, 5q33.2, 5q11.2, 5q33.3, 5p15.31, 6p21.1, 7q21.3, 5q31.2, 6p21.1, 7q21.3, 5q31.2, 5q32, 6q24.2, 7p22.3-p22.2. Losses common to seven tumors: 1p35.1, 1p33, 10q35.2, 11p11.2, 11q22.1, 11q23.1-q23.2, 20p12.2, 20p12.1
^96^	5 (3,2)	Average SCNAs was 17.3, samples had more gains than losses. Gains in four to five cases: 2ptel, 2p22.1-p22.3, 4p15.3, 5p13, 7p12.1-p12.3, 7q21-22, 18q11.2. Losses in four to five cases: 5q31.1, 8p22, 10.15, 16q22.1, 16qtel, 17ptel, 19q13.32. No clear differences NF1 vs. non-NF1.
^99^	5 (3,1,1*)	Gains seen in 2 or 3 MPNSTs: 1p36.11-1p34.2, 2q35-q37.2, 2q37, 12p11.2, 17q24-q25.3, 19p13.2-p13.3, 19p13.12-q12, 20q11.2-q12. Losses seen in two or three MPNSTs: 9p23-24, 13q12.3-q14.3, 16q12.1, 21q21.
^100^	38 (23,15)	Utilized SNP array to identify SCNAs, and characterized SCNAs were listed by gene names and corresponding cytogenetic bands. Gains in at least 21% of tumors: 8q22.1, 7p11.2, 17q25, 7q21.1, 7q31, 5p15.33, 4q11-q13, 2p25.3, 7p21.1, 8q24.21, 19q12, 12p12.1, 12p13.33, 12q14.1. Losses in at least 18% of tumors: 9p21.3, 17q11.2, 11q22.3, 13q14.2, 3p26.1, 20p12.1-pter, 5q33.2-qter, 9p22.3, 17p13.1, 9p23-24.1, 10q23.31, 22q12.2, 18q21.1. *TP53* deletion (in half of cases) was not associated with survival.

*Thought to be a radiation-induced MPNST in person without NF1.

No recurrent chromosomal translocations have been identified in MPNSTs, indicating lack of common driver fusion gene products, although unique fusions could be oncogenic in individual cases. Overall, SCNAs do not appear to differentiate sporadic versus NF1-associated MPNST, whereas some studies found minor differences between the two types (e.g. gain of 4q more common in sporadic than NF1 MPNST).^18,19^ Nearly all aCGH studies reported substantially more SCNA gains than losses in MPNSTs, suggestive of greater oncogene influence in MPNST. The gains were typically 2–4 times of the diploid signal (e.g. *CMYC* in 8q).^20^ Much less common were higher level amplifications, such as seen at 12q in 2/7 MPNSTs.^21^ A few studies evaluated the relationship between cytogenetic abnormalities and clinical outcomes. Examples include: poorer survival in tumors with gains of both 7p15-21 and 17q22-qter^22^; increased risk of tumor recurrence with 8q gain^20^; poorer survival with 8p23-p12 gain^21^; and poorer prognosis in presence of the *CDKN2A/B* deletion.^23^

Sex chromosome aneuploidies, commonly reported in MPNSTs, may correlate with tumor progression. From 103 MPNST karyotypes in the 2008 review, a third of those from males showed Y chromosome loss, whereas half of female MPNSTs showed loss of an X (a few carried partial X deletion).^15^ A few male near-triploid MPNST karyotypes also had loss of the X. Somatic loss of the Y chromosome in leukocytes is an age-related phenomenon in male humans, with greater levels of Y loss associated with increased risk of all cancers.^24^ One study reported that the only chromosomal abnormality in an MPNST was deletion of the Y (45,X,-Y).^25^ Patient-derived models suggest that sex chromosome aneuploidy may correlate with tumor behavior. Orthotopic xenografts of NF1 MPNST cell line sNF96.2, bearing a deleted Y chromosome, grew substantially faster in female mice, suggesting a possible role for steroid hormones.^26^ Thus, sex chromosome complement could play a role in MPNST behavior.

## Candidate Gene Studies

The lack of consistent involvement of specific chromosome regions was consistent with multiple and variable genetic steps in MPNST evolution. Researchers first began Sanger sequencing of known tumor suppressor genes and oncogenes in the SCNA-altered/translocated regions, to search for MPNST driver mutations. Early studies of single nucleotide variants (SNVs) or SCNAs at individual loci in MPNSTs, and subsequent protein-level studies, also showed heterogeneous results. Such studies prior to 2008 are well described in the review cited above, with the most commonly studied loci being *NF1* (17q), the p53 pathway (*TP53* (17p), *MDM2* (12q)), the pRB pathway (*CDKN2A/B* (9p), *RB1* (13q), *CDK4* (12q), *CCND2* (12p), *PTEN* (10q)), the EGF pathway (*EGFR* (7p), *ERBB2* (17q)), and growth factors/receptors (*PDGFRA* (4q), *PDGFRB* (5q), *KIT* (4q), *NRG1* (8p), *TOP2A* (17q), *MET* (7q), and *CD44* (11p)).^15^ Intragenic SNVs were only found in *NF1, PDGFRA*, *TP53*, and *NRG1*; all other changes found were SCNAs.^15^

Somatic loss of either *TP53* or *CDKN2A* is essentially invariable in MPNSTs. Molecular studies have reported *TP53* tumor suppressor abnormalities (SNVs or SCNAs) in 40–75% of MPNSTs, with biallelic losses rare.^27^ Strong p53 immunostaining of tumor sections has been correlated with *TP53* SNVs and poor survival.^28,29^*MMP13* (11q22) was of interest as a possible marker of early transformation because of increased protein expression^30^; this is consistent with increased copy number in some aCGH studies,^31,32^ but is inconsistent with observation of genetic loss in Yang and Du,^19^ highlighting the heterogeneity within these tumors. As discussed below, somatic loss of *CDKN2A* is also a common feature of MPNSTs. One group found *CDKN2A* loss (mostly homozygous) in 81% of MPNSTs,^33^ and another group found heterozygous or homozygous *CDKN2A* loss in 63%.^34^

Previous findings of increased *PDGFRA* expression in MPNST, and interest in *KIT* due to observation of mast cell infiltrates, led to testing for activating mutations or amplifications of these oncogenes. However, such mutations were rare, suggesting that any increased activity from these genes was at the transcriptional level.^35,36^ Loss of tumor suppressor *PTEN* (10q23) has also been implicated, consistent with decreased PTEN protein immunostaining of MPNST sections^37^ and genetic mutation in some MPNSTs.^38,39^ Several specific analyses of oncogene *EGFR* (7p11), involved in multiple proproliferation pathways, found increased expression in many MPNSTs (including cell lines 88-3, 88-14 and 90-8, Table 1),^40,41^ especially in association with high-grade tumors and p53 IHC-positive tumor cells.^42^ While there was no evidence of *EGFR* amplification or mutation in early studies,^43,44^ later studies did find copy-number gains at this locus in some MPNSTs (Table 2) or specific SNVs.^45^

Several studies examined specific genes through targeted sequencing. A study sequencing 151 genes in seven NF1 MPNSTs identified recurrent SNVs in *NF1* (7/7), *TP53* (2/7), *ROS1* (2/7), and *TYK2* (2/7).^45^ Multiple non-recurrent SNVs were found (e.g. *EGFR, PDGFRA, RB1, FLT1/3/4, APC, ATRX, ALK, RAF1, SMARCB1*, and *ERBB4*). Another study reported frequent inactivating mutations in *TSC1/2* genes, activating mutations in *NOTCH1* and *VEGFR2* and mutations perturbing JAK–STAT, TNF, and NFKB pathways.^46^ In another study of two MPNST cohorts, somatic *BRAF* mutations were reported in 6.5%–11.9%, with 47% of tumors having alteration in at least one *RAS/RAF* pathway gene (not including *NF1* or *BRAF*); 46% had alterations in the PI3K pathway, with 70% having alterations in at least one of these two pathways and 70% had an alteration in DNA repair genes.^47^

## RNA Expression Analysis

Early studies of MPNST candidate gene mRNA expression found different signatures. Subsequent larger studies also had some differences but consistently found some key changes common to many MPNSTs, some with correlations to clinical endpoints. Differences between study results can be explained by variables common to all approaches. Differences in experimental design included: comparing MPNSTs to vastly different controls such as neurofibromas or Schwann cells; use of primary tissue versus cultured cells; differences in gene expression platform choice; number of replicates; normalization technique; and statistical approaches. Further variables include: use of NF1-related versus sporadic MPNSTs, differences in validation, and underlying tissue heterogeneity (due to tumor stage, contaminating non-tumor tissue, patient genetic background, and variable somatic changes). Although most studies report the statistically significant observations using mean results, the data from individual tumors show substantial heterogeneity at many loci, including those that are statistically significant. Selected primary results of microarray-based RNA expression studies (candidate gene and genome-wide) are shown in Table 3, with some specific findings mentioned below.

**Table 3.** Select MPNST RNA expression array data (>400 genes screened)

Reference	Comparison	Subset of increased genes	Subset of decreased genes
^48^	16 MPNST primary vs. neurofibromas [636 chr 17 genes]	*TOP2A, FKBP10, MFAP4, VMP1, AP2B1, TIMP2, MRC2, PYCR1, MYH1, SOX9*	n/a
^49^	20 MPNSTs; NF1 vs. non-NF1 [8100 genes]	*MBP, S100B, NCAM, L1CAM, GAP43, SOX10, CRYAB*	*MKI67, FGFR1, CCNB2*
^49^	20 MPNSTs, EGFR+ vs. EGFR− [8100 genes]	*EGFR, HSF1, MFAP, COSF1, MKI67, FGFR1, BIRC5, LMNB2*	n/a
^50^	8 NF1 MPNST cell lines and 45 primary MPNST vs. normal SC [47 000 genes]	*SOX9, TWIST1, TNNT1, ADA, FOXD1, RGS2, FLRT2, PLCB2, BASP1, PAIP1, TM4SF1, RGS2, ETV1, HMGA2*	*SOX10, SPP1, CD59, TGFBR2, TIMP2, CDH19, GFRA2, PMP22, NGFR, GSN, LMNB2, SPARC, TRAF1, BHLHB2, ITGA6, ERBB2, L1CAM, HYAL1, TIMP3, CRYAB, CNP*
^44^	1 primary and 2 MPNST cultures vs. neurofibromas [539 genes]	*EGFR, HSPCA, LAMP2, MALAT1, MYO10, SEC3L1, CYP1B1*	*ITGA6, GSN, CRYAB, MATN, CD59, CDH19, RPL3, FOXD3, SPARC, APOD*
^53^	9 MPNST (mostly non-NF1) vs. 4 neurofibromas [886 genes]	*BIRC5, TNC, ADA, COL6A3, COL7A1, KRT18*	*IGFBP6*
^54^	4 MPNST vs. 6 plexiforms [16 840 genes]	*TNC, CNNB2, DNMT3A, CCND2, SEMA3A, OSF2, BIRC5, SPP1, TOP2A*	*TNXB, FOX01A, ITGB4, IGF1, TPSB, HSPB2, CBX7, GPR56*
^51^	sNF94.3 & sNF02.2 NF1 MPNST cell lines vs. normal SC [96 genes]	*VEGFC, NRP1, EGFR, ANGPT1, FGFR3, FGFR2, ADAMTS1*	*SPP1, PDGFA, ITG5A, SPARC*
^55^	11 MPNST cell lines and 6 primary MPNST vs. normal SC, but not similarly altered in neurofibromas (22 cultures and 26 primary) [47 000 genes]	*EYA4, FOXE1, FZD2, PAX6, SOX11, COL4A6, SEMA6B, ZIC1, CAST1, IHPK2, DNMT3B, HMGA2, DMRT1, EPHB2, H2AFY2, HIST1H2BD, DNER*	*S100B, ITGB3, NGFR, ERBB2, CDKN2A, PDGFRA, PDGFB, PMP22, GSN, FYN, CRYAB, SPARC, TIMP3, MIA, PSMAL, PTPRE*
^55^	Altered similarly in most neurofibromas also	*SOX9, FGFR1, EN1, EGFR, SHC1, KIT, ROBO1, CD36, S100A4, SPRY1, TWIST1, ITGB5, TNNT2, RORA, SPRY1, SPRY4, ETS2*	*CENTA1, DNMT2, GIT1, GAP43, L1CAM, MBNL2, MBP, PSD2, S100A1, TRPM3, MAF, QK1, SOX8, ITGB8, PAX3, NTRK3*
^55^	MPNST cell lines only vs. normal SC	*BMP4, COL4A5, FBLN1, MDK, MSH6, PAX6, PDE9A, S100BP, SEC14L1, EFNB2, ZIC1, AURKA, TRGB4, HGF*	*ERBB2, PMP22, GSN, ITGB3, PDGFB, TLN2, ECM1, RASSF1, CRYAB, SPARC, TIMP3, FYN, CD74, CRK, RAB7*
^57^	20 MPNST vs. neurofibromas [28 000 genes]	*BEX1, CPA3, DLK1, CRABP1, IGFBP2, IFG2, PTK7, FGFR1, TWIST1, CCNB2, GAS1, EGFR, TPX2, CYTL1*	*SOX10, MAL, CRYAB, L1CAM, ITGA2, CDH1, S100B, ERBB3, PMP22, GBP2, ITGB8, QKI, TGFBR2, TMEM23, THBS2*

n/a, not applicable; SC, Schwann cells.

Correlations between expression patterns and clinical features or tumor biology have been reported. Increased *TOP2A* (topoisomerase II alpha) expression in 10/16 MPNSTs (half NF1-related) correlated with poorer survival.^48^ An initial study of 8100 genes in 20 MPNSTs found no significant differences in expression profile based on NF1 status (10 NF1-related, 10 sporadic), although 6 genes showed a trend near significance, 3 genes were related to whether tumors were peripheral (on a limb) versus central (trunk/neck/head), and metastasis moderately correlated with expression of 12 genes.^49^ The authors then examined 22 additional MPNSTs, and found a significant signature that differentiated 9 MPNSTs (8 NF1, 1 sporadic) from the others, characterized by higher expression of neuroglial and Schwann cell differentiation genes (e.g. *MBP*, *S100B*, *SOX10)* and lower expression of several proliferation-promoting genes; none of these 9 tumors expressed *EGFR*, and appeared less differentiated histologically.^49^ In 2006, a genome-wide study identified a 159-gene profile distinguishing MPNST cell lines from wild-type Schwann cells, validated in primary MPNSTs.^50^ Schwann cell differentiation markers such as *SOX10* and *NGFR* were down-regulated, whereas *SOX9* and *TWIST1* (neural crest stem cell markers) were overexpressed. Thus, MPNST cells shared some transcription characteristics with Schwann cell precursors.^50^ A study comparing two MPNST cell lines to a normal human Schwann cell culture on a 96-gene array found that *SPP1*, *PDGFA*, *ITG5A*, and *SPARC* were significantly decreased in both cell lines, and *VEGFC*, *NRP1*, and *EGFR* were significantly increased.^51^ A later study validated increased expression of *SPP1*, along with other genes whose SCNA gains/losses corresponded to expression.^52^

Some studies have compared MPNST to neurofibroma as well. One group found 57 genes that distinguished MPNST (1 primary tumor, 2 cell lines) from cutaneous and plexiform neurofibroma (including cultured tumor cells), but interestingly, one MPNST cell culture did not closely match its primary tissue profile.^44^ A limited gene study compared 9 primary MPNSTs (1 NF1) to 4 neurofibromas (2 NF1); among genes upregulated in MPNST were *BIRC5*, *TNC*, *ADA, COL6A3,* whereas *IGFBP6* was reduced.^53^*TNC* was found upregulated in another array study compared to plexiform neurofibromas.^54^ An extensive study comparing cultured normal Schwann cells to primary tissue and cultured cells from NF1 cutaneous neurofibromas, plexiform neurofibromas, and MPNSTs found consistent changes in MPNST relative to normal Schwann cells and the benign tumors.^55^ Genes up-regulated only in MPNSTs compared to Schwann cells were related to chromosome organization, extracellular matrix, morphogenesis, and nervous system development (e.g. *EYA4*, *FOXE1*, *FZD2*, *PAX6*, *SOX11*, *COL4A6*, and *AURKA*; the latter a potential therapeutic target).^56^ Genes up-regulated in MPNST and neurofibromas compared to Schwann cells represented a variety of pathways and functions (e.g. *SOX9*, *FGFR1*, *EGFR, KIT*, *CD36*, *SPRY1*, *TWIST1*, *ITGB5)*.^55^ Those down-regulated in MPNST and neurofibromas were similarly from multiple pathways (e.g. *DNMT2*, *GAP43*, *L1CAM*, *MBNL2*, *MBP*, and *TRPM3*). There were some genes with altered expression only in the MPNST cell lines (e.g. up-regulated *BMP4*, *COL4A5*, *FBLN1, MDK, MSH6, PAX6*; down-regulated *ERBB2, PMP22, GSN, ITGB3, BCL2, PDGFB, RASSF1, SPARC, TIMP3)*. Interestingly, the analysis found no signature differentiating cutaneous from plexiform neurofibroma, but neurofibromas fell into two classes (plexiform and cutaneous in each) whose basis for differences was not clear.^55^

Identifying a specific signature for MPNST could facilitate both preclinical research for effective treatments, and clinical care in the form of molecular diagnostics to mark the transition from benign to malignant. A 2010 gene expression study of 20 MPNSTs versus neurofibromas dichotomized the MPNSTs (15 and 5), and found many more genes with reduced expression in the MPNSTs (e.g. *SOX10*, *CRYAB*, *L1CAM*, *ITGA2*, *S100B*, *ERRB3*, *PMP22*).^57^ Genes showing increased expression across all/most MPNSTs included *IFG2*, *PTK7*, *FGFR1*, *TWIST1*, *GAS1*, and *EGFR*.

A different type of approach that included knockdown of *NF1* in human Schwann cells,^58^ or a forward genetic screen^38,59^ have shown activation of WNT-signaling in the cells^58,59^ and acquisition of transformed properties.^59^ Another group has shown that in mice, ablation of *Lats1/2*, the negative regulator of Hippo-Taz/Yap axis, resulted in hyperactivation of the pathway and highly increased the animals’ susceptibility to developing low- and high-grade peripheral nerve sheath tumors.^60^

Currently, there is no consistent gene expression signature for MPNSTs, although altered expression in a few specific genes has been consistently reported, such as *SOX9/10*, *TWIST1*, *EGFR*, *FGFR1*, and *ERBB2/3*. In addition, several pathways and processes, including oligodendrocyte specification, neural crest differentiation, focal adhesion, RAS-MAPK-, PI3K-Akt-mTOR-, WNT-, and Hippo-Merlin-signaling appear to be affected with increased frequency.

## Epigenetic Analyses

As with expression signatures, epigenetic alterations could serve as a molecular diagnostic marker. Several studies have implicated variable epigenetic alterations in MPNSTs, with most examining cytosine methylation at CpG dinucleotides in gene promoter regions, through candidate genes or genome-wise approaches. A 1999 study found no evidence of hypermethylation at the *CDKN2A* in 11 NF1-related MPNSTs even in tumors with intact 9p21, suggesting that methylation is not a common mechanism of gene silencing in that region.^61^ A later study of 3 MPNSTs found evidence of variable methylation at the CpG islands of 11 tumor suppressor genes (normally unmethylated), including 19% methylation at *CDKN2A*, *RB1*, *p14*^*ARF*^, and *THBS1* in one tumor; *MGMT* in another; and *TIMP3* and *THBS1* in the third one.^62^ A 2006 study examined 17 MPNSTs (5 NF1-related) and found aberrant CpG methylation at 0–5 loci (most having just 1 gene affected) with the two most commonly involved being *DAPK* and *PTEN* (5 tumors each).^63^

Feber et al.^64^ used an array for the first genome-wide methylation study in MPNST, finding hundreds of cytosines at CpGs differentially methylated between neurofibromas and MPNSTs. Several dozen of the correlated genes were among those previously reported to be differentially expressed such as *S100B* and *SOX10*,^55,57^ but *CDKN2A* was not found significantly differentially methylated. In a genome-wide DNA methylation study of 10 MPNSTs and other sarcomas in 2013, although seven tumors failed to cluster among themselves (unsupervised analysis) or with other sarcomas based on overall methylation profile, the authors were able to identify a characteristic set of 38 methylated cytosines for MPNSTs with further analysis.^65^ The three most significant cytosines were in the *FBLN2*, *MICALL2*, and *EFCAB1* genes respectively, and overall the study found hyper-methylation at the subgroup-specific cytosines. The two MPNST cell lines analyzed (STS26T and T265) yielded similar results to the primary tissues. A study of tumors from the same NF1 patient at the *TAGLN* locus found that only the MPNST lacked methylation, correlating with increased expression of transgelin,^66^ but this is in contrast to another study that found decreased expression relative to neurofibromas.^57^

De Raedt et al.^67^ used multiple models to show that loss of *SUZ12* was common in NF1 MPNSTs, and it amplified the RAS downstream effects through chromatin remodeling (see a recent review summarizing data for inactivation of PRC2 in MPNST^68^). A 2015 study followed up on one of Feber’s findings^64^ to examine methylation status of the *RASSF1* tumor suppressor gene promoter in a large series of NF1-related and sporadic MPNSTs.^69^ They found that overall, 60% of MPNSTs showed hypermethylation at *RASSF1*, and it was a poor prognostic marker when present in tumors from patients with NF1. Lee et al.^33^ found loss of PRC2 function (through *SUZ12* or *EED* mutation) in over 70% of MPNSTs (regardless of NF1 disease status), and showed that this resulted in loss of H3K27 trimethylation (H3K27me3), a repressive epigenetic histone modification, which correlated with transcriptional alterations in 479 genes, such as upregulation of *IGF2*, *PAX2*, and *TLX1*. A 2016 study screened 162 MPNSTs for H3K27me3 by immunohistochemistry, finding loss in 34% of MPNSTs, compared to none in neurofibromas.^70^ Patients with tumor loss of H3K27me3 also had a poorer prognosis.

An epigenome study of MPNST and neurofibromas found that the methylome was different between high- and low-grade MPNST, the latter being indistinguishable from atypical neurofibroma.^71^ High-grade MPNSTs fell into two epigenomic subgroups regardless of neurofibromin status, correlating with presence of H3K27me3. Of interest, the high-grade MPNSTs retaining H3K27me3 all originated from spinal nerve roots. A later epigenetic study examined global changes in CpG methylation, 5-hydroxymethylcytosine, and H3K27me3 in 8 MPNSTs and 20 neurofibromas.^72^ MPNSTs had decreased H3K27me3, but there was only borderline decreased global CpG methylation compared to the benign tumors, unlike the Renner study.^65^ These studies support a role for this mechanism in aberrant gene regulation in MPNST, and further characterization of these marks in MPNSTs may provide diagnostic and prognostic information.

## Next-Generation Sequencing Studies

More recent studies have leveraged the availability of exome and genome sequencing, in addition to expression profiling and methylation analysis, to provide a more thorough view of the MPNST genomic landscape. Three papers published in 2014 were the first to investigate MPNST molecular architecture using these methods. Lee and co-authors (2014)^33^ found frequent somatic alterations in *NF1* (72%), *CDKN2A* (81%, mostly homozygous loss), *SUZ12*, and *EED*. Moreover, these alterations significantly co-occurred, implying cooperativity in malignant transformation. Besides mutation of *TP53* (47%), no other recurrent mutations were observed. These findings were in agreement with the work of De Raedt et al.,^67^ who also observed frequent inactivation of PRC2 in MPNST. They investigated *SUZ12* and *EED* status in 19 MPNSTs with germline *NF1* microdeletion and 39 MPNSTs with other *NF1* germline mutations. PRC2 function was abrogated in 79% of tumors with *NF1* microdeletion and in 34% of the others. Another study in 2014, which utilized exome, genome and gene-panel sequencing of 50 MPNSTs, similarly found inactivation of *SUZ12* in 16 MPNSTs.^73^

An exome analysis of eight NF1 MPNSTs found uniform inactivation of *NF1*; heterozygous or homozygous *CDKN2A* deletion in 63%; and *SUZ12* or *EED* mutations in 87.5% of samples.^34^ Homozygous *TP53* deletion was found in one MPNST, and another harbored a heterozygous missense mutation in *KDM2B* (a master regulator of polycomb complex PRC1) that was predicted to be deleterious. Expression studies of another set of 14 MPNSTs also found reduced *KDM2B* mRNA levels compared to neurofibromas. The overall number of somatic mutations in MPNSTs was considered low (median 41 per tumor) and most mutations were nonrecurrent. Another project combined exome sequencing of five MPNSTs (four NF1-related) with prior exome data of seven other MPNSTs, observing frequent alterations in *NF1* (92%), *CDKN2A* (58%), PRC2 (*SUZ12* (42%), *EED* (33%)), and *TP53* (50%).^74^ They identified no other recurrent mutations, but six tumors had somatic alterations at one or more of six RAS signaling genes: focal amplification of *KIT* and *PDGFRA*, activating missense mutations in *PIK3CA*, *PTPN11*, and *FGFR1*, and a heterozygous truncating mutation in *RASSF9*. In addition, they noted likely inactivating mutations in cell cycle genes *RB1* (two tumors) and *CHEK2* (one). The median tumor mutation burden was 63.

A set of soft-tissue sarcomas including five MPNSTs was evaluated with an integrated genomic approach by The Cancer Genome Atlas.^75^ The median somatic mutations per MPNST was 50 (vs. 100 for all sarcomas), and the median SCNAs per MPNST was 250 (vs. 200 for all). Seven genes were significantly frequently mutated in MPNSTs: *NF1* (2/5), *TP53* (1/5), *DICER1* (1/5), *ARID1A* (1/5), *FGFR1* (1/5), *KIT* (1/5), and *APC* (1/5). *CDK4, MDM2*, *CCNE1*, and *TERT* were among the genes with gains, and *CDKN2A*, *RB1*, and *NF1* were frequently deleted in the tumors. Of note, no somatic mutations/alterations in *SUZ12* or *EED* were reported in this study. Exome sequencing has also helped to elucidate genetic events underlying the transformation from plexiform neurofibroma to atypical (ANF) to MPNST. A recent study found that two ANFs from a single patient had heterozygous and homozygous *CDKN2A/B* deletion plus bi-allelic *NF1* mutation.^76^ The *CDKN2A/B* locus copy-number correlated with histological features: the classic ANF carried a single copy loss, whereas the other ANF had both alleles deleted. The authors concluded that transformation to a premalignant state is driven by increasing *CDKN2A/B* deficiency, and the tumor with the homozygous deletion defined a category now called “atypical neurofibromatous neoplasms of uncertain biologic potential” (ANNUBP). In mice, conditional ablation of *Nf1* and *Cdkn2a* in Schwann cell lineage results in tumors that phenocopy human ANNUBP and frequently progress to MPNST.^77^ Another genomic study found that most ANFs have normal diploid genomes, low-somatic mutation load, and frequent *NF1* inactivation and SCNAs in *CDKN2A/B* and *SMARCA2*.^8^ Importantly, no mutations, SCNAs or changes in expression were detected in *SUZ12, EED*, or other PRC2 genes, in contrast with that published in most MPNSTs. Next-generation sequencing studies have revealed that the genomic architecture of MPNST resembles that of other soft tissue sarcomas, with a relatively modest burden of point mutations and an elevated number of SCNAs as their most distinguishing genetic features. Genes bearing rare deleterious MPNST somatic mutations predominantly clustered in several signaling pathways, including RAF-MEK-ERK and PI3K-AKT-mTOR. These studies suggest that in NF1-associated MPNST (vs. sporadic or radiation-induced), development of the fully malignant state commonly involves the following steps: (1) tumor initiation following somatic loss of the second *NF1* allele; (2) loss of *CDKN2A/B* resulting in premalignant ANF/ANNUBP; and (3) loss of PRC2 function initiating malignancy. Transition from the benign to malignant state also coincides with rise of genomic instability, which likely accelerates loss of function of key tumor suppressors (e.g. *EED*, *SUZ12*) and affects copy-number status of multiple oncogenes and other tumor suppressor genes.

## Conclusions

Improvement in clinical care is the ultimate goal of understanding genetic drivers of MPNST initiation and progression. The past 15 years have seen tremendous progress in the state of MPNST research, but this is tempered by the realization that each incremental advance in our collective knowledge opens a plethora of additional questions. As with other areas of cancer genetics, the level of MPNST genomic complexity is tremendous. Studies have shown that a substantial level of MPNST heterogeneity is due to a high degree of genomic instability (reflected at the chromosome and copy-number levels). Beyond the static genome, mRNA expression analysis and epigenetic profiling are helping identify important aspects of MPNST biology, and have validated mechanisms in aberrant signaling. Mutations and copy number changes in *NF1*, *CDKN2A/B*, *SUZ12*, *EED*, and/or *TP53* are found in most MPNST regardless of NF1 diagnosis; however, other signaling pathways have been implicated. Like most other soft-tissue sarcomas, MPNSTs carry a relatively low burden of point mutations but elevated SCNV load. It appears that at least in NF1-associated MPNSTs, progression from benign to malignant status frequently proceeds through sequential inactivation of three tumor suppressors: *NF1*, *CDKN2A/B*, and *SUZ12* and/or *EED* (PRC2 complex). Despite substantial genomic heterogeneity of MPNSTs, their transcriptomes, in at least a subset of tumors, converge on a profile resembling immature Schwann cells. Frequent ubiquitous loss of H3K27 tri-methylation reflects PRC2 inactivation and global reprogramming of gene expression through epigenetic regulatory mechanisms, a feature that may relate to prognosis. Future challenges include single-cell deep DNA and RNA sequencing to better understand clonal and tumor–stromal–immune system architecture in MPNSTs. Comprehensive genomic profiling of many more individual MPNSTs will be indispensable to understanding the full range of molecular diversity of MPNSTs. However, a full understanding will not be achieved until we move beyond bulk tissue and cell lines, to leverage technologies such as single cell DNA/RNA sequencing and epigenetic profiling. We foresee these approaches as the best hope for a giant leap forward that will facilitate impactful progress in designing and testing targeted, personalized therapeutic strategies for MPNST.

## Funding

This work was supported by Children’s Tumor Foundation, and the Neurofibromatosis Therapeutic Acceleration Program acknowledged by M.R.W. And from the Neurofibromatosis Research Initiative, made possible by an anonymous donation to Boston Children’s Hospital acknowledged by D. T. M.
